# Therapeutic Promises of Bioactive Linarin, a Glycosylated Flavonoid: A Comprehensive Review With Mechanistic Insight

**DOI:** 10.1155/jotm/9989759

**Published:** 2025-10-12

**Authors:** Faysal Mollah, Mst Muslima Khatun, Raihan Chowdhury, Md. Shimul Bhuia, Jeba Anika Sultan, Sharmita Ghosh Situ, Md. Sakib Al Hasan, Hossam Kamli, Muhammad Torequl Islam

**Affiliations:** ^1^Department of Pharmacy, Gopalganj Science and Technology University, Gopalganj 8100, Bangladesh; ^2^Phytochemistry and Biodiversity Research Laboratory, BioLuster Research Center, Gopalganj 8100, Dhaka, Bangladesh; ^3^Department of Clinical Laboratory Sciences, College of Applied Medical Sciences, King Khalid University, Abha 61421, Saudi Arabia; ^4^Pharmacy Discipline, Khulna University, Khulna, Bangladesh

**Keywords:** flavonoid glycoside, linarin, molecular mechanism, pharmacological effects

## Abstract

Linarin, a flavonoid glycoside found in the Asteraceae, Lamiaceae, and Scrophulariaceae families, shows diverse therapeutic benefits in laboratory studies. This study aims to evaluate the pharmacological effects of linarin through clinical and preclinical experiments, investigating its mechanisms against different diseases. To achieve this, data collection and search operations were carried out (current as of April 09, 2024) in several reliable online databases, such as PubMed/Medline, Scopus, Springer Link, ScienceDirect, Wiley Online, Web of Science, and Google Scholar, as part of this research. Studies revealed that linarin provides benefits against inflammation and various diseases, including osteoporosis, osteoarthritis, liver injuries, diabetes, hypertension, and neurodegenerative conditions, such as Alzheimer's, ischemia-reperfusion, convulsions, and depression. Our results further reveal that linarin displays remarkable anticancer potentials through multiple molecular pathways, including apoptotic cell death, oxidative stress induction, cytotoxic effect, antiproliferative effect, genotoxic effect, and mitigation of cancer cell migration and invasion and migration against a range of malignancies, including lung, brain, prostate, and glioma cell cancers. Database reports and the current literature-based study suggest that linarin might be a prospective lead chemical for treating a range of illnesses and ailments. For linarin to be proven as a medicine, further clinical and preclinical trials are required.

## 1. Introduction

Natural products (NPs) are biologically active substances derived from natural sources such as plants, animals, and microbes [1]. Herbal remedies and folklore medicines have been used for a very long time to treat a wide range of diseases, both in developed and developing countries. In rural and impoverished areas, the majority of people have a strong belief in herbal remedies, medicinal plants, and home treatments. These alternatives are often more cost-effective than pharmaceutical medications [2, 3].

Around two centuries ago, the first pharmacologically potent compound isolated from a plant was morphine, obtained from poppy (Papaver somniferum) and discovered by a novice pharmacist (age 21) named Friedrich Sertürner. This ushered in a new era, in which medications derived from plants could be refined, analyzed, and delivered [4]. The discovery of penicillin in a mold culture by Alexander Fleming in 1928 indicated the beginning of the antibiotic era as we know it today [5]. This finally transformed the field of drug discovery research [6]. When the field of antibiotic discovery was at its peak in the 1940s–1960s, most antibiotics were first identified via the screening of actinomycetes that were acquired from soil [7]. One of the main sources of innovative structural leads will always be found in nature [8]. NPs have been utilized to discover anticancer medications, including vinblastine from Catharanthus roseus and taxol from Taxus brevifolia. Additionally, antimalarial drugs such as artemisinin from Artemisia annua and quinine from Cinchona species originated from plant sources and have been shown to be effective in treating a variety of ailments [9]. Digitalis fights against heart failure and some irregular cardiac rhythms [3]. The chemical variety of natural commodities matches the chemical diversity of effective medications better than the diversity of collections of synthetic molecules [10].

Additionally, compared to fully synthesized substances, NPs are more likely to mimic biosynthetic intermediates or endogenous metabolites and hence benefit from active transport pathways, and compared to manufactured medications, natural substances are more easily absorbed [10, 11]. Nowadays, chemical drug synthesis has failed to meet expectations despite estimates. For instance, of the 1135 novel medications registered between 1981 and 2010, only 36% were fully synthetic, and the remaining compounds were analogues, derivatives, or derived from natural sources. The previously mentioned statistics prompted the researchers to regain their interest in studying NPs [1].

Phenolic compounds are associated with various medicinal plant benefits due to their anti-inflammatory, anticancer, antibacterial, and antioxidant properties [12]. One of the greatest naturally occurring phenolic compounds with several beneficial biological features has been identified among the various phytochemical categories as flavonoids. Flavonoids are members of the phytochemical class known as polyphenols. Polyphenols were formerly used as treatment in Ayurvedic and Chinese medicine. These substances are grouped into six primary divisions according to their chemical composition: flavones, flavanones, flavanols, flavan-3-ols, isoflavones, and anthocyanins [13, 14]. Flavonoids exhibit powerful antioxidative, anti-inflammatory, antimutagenic, antimicrobial, anticarcinogenic, and vascular actions. Several enzymes including aldose reductase, Ca^2+^ ATPase, XOs, COXs, lipoxygenase, and phosphoinositide 3-kinase, are potently inhibited by them [14]. Recent studies have highlighted the multifaceted therapeutic potential of flavonoids, particularly their roles in modulating apoptosis, autophagy, and immune responses [15, 16]. Flavonoids have been shown to influence key signaling pathways, such as the NF-κB and MAPK pathways, leading to the regulation of inflammatory cytokines and apoptotic proteins. These actions contribute to their anticancer, neuroprotective, and anti-inflammatory effects [17]. Their immunomodulatory effects further support their potential as adjunctive therapies in managing immune-related disorders.

**Figure 1:**
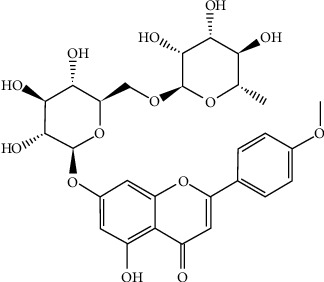
Chemical structure of linarin.

Linarin (acacetin-7-O-rutinoside) (Figure 1), a natural flavonoid glycoside, has been found in a variety of plant species, mostly from the Asteraceae, Lamiaceae, and Scrophulariaceae families, specifically from Cirsium, Micromeria, and Buddleja species [13]. Linarin, found in Chrysanthemum indicum [18], has received attention as a potential natural substance because of its wide range of pharmacological effects such as analgesic, antipyretic [19, 20], anti-inflammatory [21], neuroprotective [22], antidiabetic [23], cardioprotective [24], osteoprotective [25], phagocytosis downregulation [26], anticancer [27], prevention of osteoblastic dysfunction [28], antihypertensive [29], sedative [30], and anticonvulsant [31] activities. Additionally, it can be utilized to manage and cure osteoporosis [32], osteoarthritis [33], diabetic liver injury [34], hepatic failure [35], and lung injury [36]. This study aims to summarize the pharmacokinetic (PK), toxicological, and clinical data of linarin alongside its biological activity.

## 2. Methodology

### 2.1. Search Strategy

The selection of the literature was done by utilizing computerized databases such PubMed, Springer Link, Scopus, Wiley Online, Nature, Web of Science, Medline, and ScienceDirect with the terms “linarin,” and then paired with “biological activities,” “osteoprotective activity,” “anti-inflammatory activity”, “antioxidant,” “oxidative stress,” “protective effect,” “tumor,” “anticancer,” “cancer,” “cytotoxic effect,” “obesity,” “hepatoprotective activity,” “cardioprotective effect,” “antimicrobial effect,” “anti-fungal,” “antiviral effect,” “pharmacological effects,” “therapeutic benefits,” “biological sources,” “neurological effect,” “anti-diabetic effect,” “cerebral ischemia/reperfusion,” “chemical features,” “physicochemical features,” “pharmacokinetics,” “in vivo studies,” or “in vitro studies.” The use of words was unrestricted. The investigation has been subjected to a thorough assessment that includes information on the data's source, dose, and concentration levels, the experimental models used, potential mechanisms of pharmaceutical actions, and overall conclusions.

### 2.2. Inclusion and Exclusion Criteria

The following criteria were used to determine which studies were eligible for inclusion: (1) studies conducted in vitro, ex vivo, in vivo, and in silico, (2) studies involving pharmacological functions, (3) research on linarin's botanical origins, (4) research with or without proposed mechanisms of action, and (5) research on the PK of linarin. Conversely, the exclusion criteria included the following: (a) studies without full-text availability; (b) papers authored in languages other than English; (c) case reports, letters, editorials, and commentaries; and (d) duplicate data, titles, and/or abstracts that did not fit the inclusion requirements.

### 2.3. Physicochemical and PK Features

PK generally examines how a medication alters during the absorption, distribution, metabolism, and excretion (ADME) processes after it is administered [37]. To prescribe a medication to a neonate, baby, child, or teenager, it is necessary to accurately determine the PKs and pharmacodynamics (PD) of that specific medicine. The volume of distribution (*V*_*d*_) and clearance (*C*_*L*_) are the two most significant PK parameters for pharmaceuticals [38]. However, the PK parameter mostly depends on the physiochemical properties of the drugs.

Linarin (C_28_H_32_O_14_) (7-{[6-O-(6-deoxy-α-L-mannopyranosyl)-β-D-glucopyranosyl] oxy}-5-hydroxy-4′-methoxyflavone) [39]. It is a white powder. This chemical has a melting point of 258°C–260°C and a boiling point of 885.2 ± 65.0°C (predicted). Linarin is a hygroscopic substance with a pKa value of 6.11 ± 0.40. The substance dissolves in methanol but is almost insoluble in ether. Linarin has poor oral bioavailability. Research data showed that the bioavailability of the liposome formulation of linarin was 0.9886 times greater than that of linarin, and linarin solid dispersion was 3.363 times greater than that of linarin. It happened due to the suppression of cellular efflux mediated by P-glycoprotein in intestinal absorption. Simultaneously, piperine markedly increased the oral absorption of linarin in rats via suppressing the metabolism of linarin [39, 40]. Following linarin solution injection intramuscularly, linarin was rapidly absorbed and attained a peak level of concentration of 323.5 ± 34.5 ng/mL within 0.33 h. In contrast, the plasma concentration was eliminated quickly, with an average elimination half-life of 1.27 h [41]. When linarin was administered intragastrically to normal rats, it was quickly absorbed and reached its maximal concentration around 7 min later. Linarin showed notable variations, suggesting that liver injury may have an impact on linarin's liver distribution mechanism [42].

## 3. Pharmacological Activities of Linarin

### 3.1. Anti-Inflammatory Effect

Inflammation is the immune system's essential response to infection and tissue damage, driven by mediators like cytokines, prostaglandins, nitric oxide, and oxygen-free radicals, which coordinate the response. These chemicals are produced by invading inflammatory cells, along with endothelial and epithelial cells. Inflammatory mediators are a double-edged sword; they help prevent infection but can also damage the host [43]. Prolonged inflammation results in several ailments that, when combined, are the primary causes of inability and death around the world, including autoimmune and neurodegenerative diseases, cancer, diabetes mellitus, chronic renal disease, nonalcoholic fatty liver disease (NAFLD), cardiovascular disease, and diabetes [44].

An in vitro study by Kim et al. [26] demonstrated that linarin exhibits anti-inflammatory effects. Linarin, at concentrations of 5, 10, 20, and 30 μM, reduced the expression of proinflammatory cytokines (IL-1β and IL-6), major histocompatibility complex class II (MHC II), nitric oxide (NO), and cluster of differentiation 80 (CD80) in RAW264.7 cell lines. Additionally, linarin at concentrations of 10 and 100 μmol/L has shown potent anti-inflammatory effects. This activity was confirmed by its ability to inhibit the expression of NO, tumor necrosis factor-alpha (TNF-α), and interleukin-1 beta (IL-1β) in lipopolysaccharide (LPS)-induced human umbilical vein endothelial cells (HUVECs) [45]. Another study showed that linarin, at concentrations of 2.5, 5, 10, and 20 μg/mL, modulates inflammation in LPS-activated RAW 264.7 cell lines. Linarin increased the levels of cytokines IL-1 and TNF while reducing NO production. As a potential novel therapeutic agent, linarin may be beneficial in treating endotoxemia and inflammation associated with excessive NO production [46]. Linarin, at concentrations of 40, 80, and 160 μM, has shown significant effects on LPS-induced RAW264.7 cells by strongly suppressing the levels of proinflammatory mediators such as NO, TNF-α, IL-1β, and IL-6. It also reduced mRNA levels and the expression of iNOS, NF-κB p65, IκBα, ERK, JNK, p38, Akt, and MAPKs. These findings suggest that linarin could serve as a potential modulatory agent for the management and prevention of inflammatory diseases [47]. Furthermore, an in vitro study found that linarin, at concentrations of 20, 40, and 80 μM, offers therapeutic potential for inflammatory diseases by reducing markers of inflammation. In LPS-stimulated RAW264.7 cell lines, linarin effectively lowered levels of NO, TNF-α, IL-6, and prostaglandin E2 (PGE2) [48]. Linarin, at concentrations of 8–32 μM, significantly inhibited the release of NO induced by LPS in macrophages. This study provides strong support for the anti-inflammatory mechanism of linarin in the context of dampness-heat stasis syndrome associated with pelvic inflammatory disease [49]. An in vivo study found that linarin, administered at doses of 40, 80, and 120 mg/kg for mice and 20, 40, and 60 mg/kg for rats, significantly reduced inflammation. It decreased xylene-induced ear edema, acetic acid-induced vascular permeability, and carrageenan-induced hind paw edema in mice, and cotton pellet-induced granuloma formation in rats. These effects were linked to reductions in the levels of iNOS, COX-2, TNF-α, IL-1β, IL-6, PGE2, and malondialdehyde (MDA), as well as increases in superoxide dismutase (SOD), glutathione peroxidase (GPx), and glutathione reductase (GRd). The study suggested that linarin exerts its anti-inflammatory effects by reducing reactive oxygen species (ROS) and inhibiting NF-κB signaling [50] (Table 1).

### 3.2. Osteoprotective Effect

A condition known as osteoporosis is defined by diminishing bone mass, worsening microarchitecture, and fragility fractures [51]. One way to think of osteoarthritis is as the pathological and clinical consequence of a number of conditions, leading to the structural and functional breakdown of synovial joints. When the dynamic balance between joint tissue deterioration and healing is upset, osteoarthritis results [52]. Osteoporotic fracture is linked to a number of risk indicators such as low peak bone mass, hormonal variables, the use of certain drugs (such as glucocorticoids), smoking cigarettes, poor physical activity, low calcium and vitamin D consumption, race, small body size, and a history of fractures in the family or individually [53]. The pathophysiology of osteoporosis has been expanded to encompass impaired skeletal fragility and bone strength due to multiple factors: (1) defects in the inherent material characteristics of bone tissue, (2) defects in the microarchitecture of trabeculae, (3) improper healing of microdamage from routine everyday activities, and (4) high rates of bone remodeling. These causes all occur in the context of age-related bone loss [54]. Almost one in three women suffers from the common clinical disorder known as postmenopausal osteoporosis. Rapid bone loss is caused by low estrogen, and it peaks in the first two to 3 years following menopause [55].

**Figure 2:**
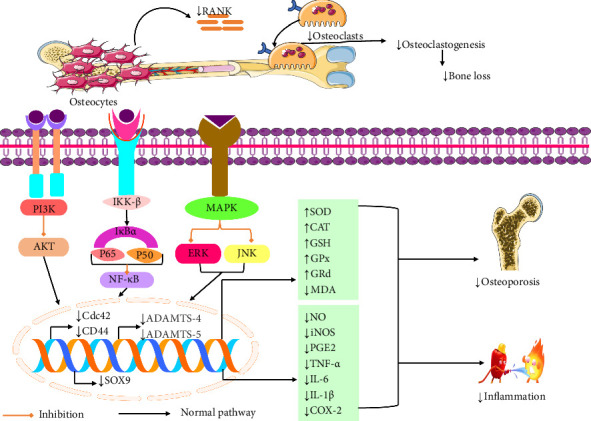
Possible mechanism of linarin against osteoporosis and inflammation [PI3: phosphoinositide 3-kinase; AKT: protein kinase B; IκBα: nuclear factor of kappa light polypeptide gene enhancer in B-cell inhibitor alpha; IKKβ: IκB kinase β; iNOS: inducible nitric oxide synthase; NF-κB: nuclear factor kappa-B; RANK: receptor activator of nuclear factor κB; P65: transcription factor P65; P50: transcription factor P50; JNK: Jun N-terminal kinase; ERK: extracellular signal-regulated kinase; Cdc42: cell division control protein 42 homolog; CD44: cell surface adhesion receptor; SOX9: SRY-Box transcription factor 9; SOD: GPx: GRd: MAPK: mitogen-activated protein kinase; GSH: glutathione; PGE2: prostaglandin E2; MRD: minimal residual disease; MDA: malondialdehyde; COX-2: cyclooxygenase 2; CAT: chloramphenicol acetyltransferase; IL-6: interleukin 6; IL-1β: interleukin-1beta; TNF-α: tumor necrosis factor-alpha; NO: nitric oxide].

An in vivo study by Yang et al. [25] found that linarin has an osteoprotective effect. Linarin, given at doses of 20 and 40 mg/kg, reduced cadmium-induced osteoporosis in mice by increasing levels of SOD, catalase (CAT), and GPx, while decreasing levels of MDA, lipid peroxidation, RANKL mRNA, NF-κB p65, IKKβ, IL-6, and TNF-α. The study suggests that linarin may treat cadmium-induced osteoporosis in mice by reducing oxidative stress and inflammation and by modulating the RANK/RANKL/OPG pathway [25]. Another investigation by Kim et al. [56] demonstrated that linarin (1–20 μM) showed osteoprotective effects on RAW 264.7 cell lines by decreasing RGD peptide, paxillin, gelsolin, cdc42, CD44, and osteoclast function. These findings indicated that linarin was successful in blocking diffuse cloud-associated αvβ3 integrin and core-linked CD44, which in turn slowed down osteoclast function of focused attachment to bone matrix and active bone resorption [56]. Furthermore, the study revealed that linarin (3.75–30 μM and 30 mg/kg) exerted its osteoprotective effect in both in vitro and in vivo (Qi et al. 92,021). Recent research demonstrated that when linarin was administered at a dose of 20 mg/kg, it reduced ovariectomy (OVX)-induced bone loss in C57BL/6 female mice by increasing αvβ3 integrins, proton suppliers, OCN, osteopontin, nonspecific alkaline phosphatase (ALP), and also reducing cathepsin K and matrix metalloproteinase-9 (MMP-9). This study suggested that linarin demonstrated efficacy in OVX circumstances by delaying osteoclastic bone resorption and stimulating osteoblastic bone matrix mineralization [33]. Linarin at concentrations of 0.1, 1, and 10 mg/mL markedly minimized RANKL (50 ng/mL)-induced osteoclastogenesis in C57BL/6 mice by alleviating NFATc1, TRAP, OSCAR, c-Fos, NF-kB p65, and NF-kB signaling pathway, leading to antiosteoclastic properties and preventing the bone loss [57]. Furthermore, both in vitro and in vivo studies demonstrated that linarin significantly attenuated osteoporosis. This effect was associated with a reduction in serum levels of ALP and osteocalcin (OCN) and an increase in osteoblast differentiation in MC3T3-E1 cells. Linarin also enhanced extracellular matrix mineralization and the expression of OCN, bone sialoprotein (BSP), collagen Type I (COL-I), and ALP. Additionally, it promoted the phosphorylation of SMAD1/5, protein kinase A (PKA), and the BMP-2/RUNX2 signaling pathway [58]. Linarin, at concentrations of 0.2–5 mg/mL, has been shown to prevent hydrogen peroxide-induced dysfunction in osteoblasts and may exert antiresorptive effects by reducing oxidative damage and RANKL levels [28]. Additionally, an in vitro study indicated that linarin has an osteoprotective effect on SW1353 cell lines, leading to reductions in the expression of SOX9, ADAMTS-4, and ADAMTS-5 [59]. A possible mechanism of linarin against osteoporosis and inflammation is displayed in Figure 2.

### 3.3. Hepatoprotective Effect

The three main diseases of the liver are alcoholic hepatic disease, nonalcoholic fatty liver disease, and viral hepatitis (mostly caused by the hepatitis B virus [HBV]) [60]. Hepatocyte infections caused by viruses can cause damage to the liver in cases of viral hepatitis [61]. Liver disease contributes to two million annual fatalities and 4% of all deaths worldwide (one death for every 25). Acute hepatitis accounts for a lesser percentage of mortality, with complications from cirrhosis and hepatocellular cancer being the main causes of death [62]. Alcoholic fatty liver may evolve into alcoholic steatohepatitis (ASH), a disorder distinguished by inflammation of the liver. Over time, chronic ASH may produce cirrhosis, fibrosis, and sometimes even hepatocellular carcinoma (HCC) [63]. About 25% of people worldwide suffer from NAFLD as Type 2 diabetes mellitus (T2DM), obesity, and metabolic syndrome increase in prevalence [64]. Metabolic illnesses like central obesity, dyslipidemia, hypertension, hyperglycemia, and chronic abnormalities in liver function tests are intimately linked to NAFLD [65]. The main factor contributing to chronic liver disease is NAFLD. A small percentage of individuals have increasing inflammation, fibrosis, and liver damage; this is known as non-ASH. The primary cause of mortality for those with NAFLD is cardiovascular disease [18].

**Figure 3:**
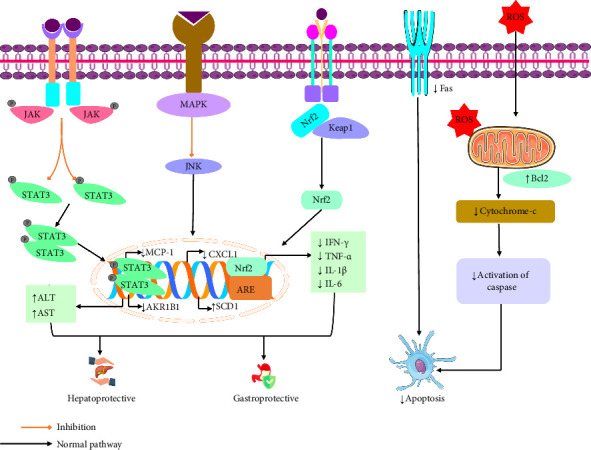
The possible hepatoprotective, gastroprotective, and antiapoptotic effects of linarin are supported by several studies in the literature [MCP-1: monocyte chemoattractant protein-1; JAK: Janus kinase; ALT: alanine transaminase; AST: aspartate aminotransferase; AKR1B1: Aldo-Keto reductase family 1, member B; CXCL1: chemokine (C-X-C motif) ligand 1; SCD1: stearoyl-CoA desaturase 1; Nrf2: nuclear factor erythroid 2–related factor 2; Keap1: Kelch-like ECH-associated protein 1; IFN-γ: interferon-gamma; ROS: reactive oxygen species; IL-1β: interleukin-1beta; TNF-α: tumor necrosis factor-alpha; IL-6: interleukin 6; Fas: fatty acid synthase; Bcl2: B-cell leukemia/lymphoma 2 protein; STAT3: signal transducer and activator of transcription 3].

An in vivo study demonstrated that linarin, administered at doses of 12.5, 25, and 50 mg/kg, attenuated GalN/LPS-induced hepatic failure in male ICR mice. This effect was associated with a decrease in Fas-associated death domain (FADD), caspase-8, cytochrome c, caspase-3, and the phosphorylation of the proapoptotic protein Bim, and the TNF-α-mediated apoptotic pathway. Conversely, linarin increased the levels of Bcl-xL and the phosphorylation of STAT3. These findings indicate that linarin reduces liver damage caused by GalN/LPS by inhibiting TNF-α-mediated apoptotic pathways [35]. Another in vivo study by Zhuang et al. [66] showed that linarin has hepatoprotective effect at dose of 15, 30, and 60 mg/kg lowered NASH-induced HFHC in male Sprague–Dawley rats via decreasing liver cholesterol, SCD1, ALT, AST, c-JNK, MCP-1, TNF-α, CXCL1, and lobular inflammation. Based on findings, HFHC diet-induced liver damage and inflammation in rats may be mitigated by linarin. Part of this protective effect might be attributed to its modulatory influence on lipid metabolism [66]. Linarin significantly attenuated acute liver injury in a CCl_4_-induced male BALB/c mouse model following administration at doses of 12.5, 25, and 50 mg/kg once daily for 7 days. The protective effects were associated with a reduction in ROS-induced oxidative stress, suppression of TLR4/MyD88 and JNK/p38/ERK-mediated inflammatory signaling pathways, and enhancement of autophagic flux via upregulation of Beclin 1 and LC3II expression [67]. An in vitro study stated that linarin at concentration of 50, 25, and 12.5 μg/mL exhibited protective effects against CCl_4_-induced hepatotoxicity on HepG2 cell lines by increasing ALT, AST, Keap1, Free Nrf2, and Phase II detoxification enzymes and by reducing ROS and MAPKs. The hepatoprotective properties of linarin are supported scientifically by this investigation [68]. Furthermore, an in vitro study demonstrated that linarin at a concentration of 20 μM, along with an in vivo study administering doses of 15, 30, and 60 mg/kg/day, mitigated hepatocyte damage caused by elevated glucose and palmitic acid in mice. Linarin achieved this by reducing hepatic lipid accumulation, oxidative stress, inflammation, and apoptosis, as well as levels of AKR1B1. The findings suggest that linarin may help prevent liver damage in individuals with T2DM by alleviating inflammation and oxidative stress mediated by AKR1B1 [34] (Table 1).  Figure 3 illustrates the protective mechanism of linarin.

### 3.4. Gastroprotective Activity

Peptic ulcers are a prevalent condition affecting the entire gastrointestinal tract, primarily the stomach and the proximal duodenum. This condition is characterized by multiple factors and its treatment encounters significant challenges because of the limited efficacy and severe adverse effects of the already accessible medications. Flavonoids exhibit several pharmacological effects in the field of gastroprotection, functioning as antisecretory, cytoprotective, and antioxidant agents. Furthermore, these polyphenolic compounds not only contribute to the healing of gastric ulcers, but they also have the potential to serve as novel options for the suppression or modulation of peptic ulcers associated with *H. pylori* [69].

A recent in vivo study of linarin showed that linarin (25 and 50 mg/kg) considerably reduced dextran sulfate sodium (DSS)–induced colitis in C57BL/6J mice via reducing inflammatory molecules IL-6, TNF-α, IFN-γ, IL-1β, and myeloperoxidase and that is improved histopathological damage, mucosal layer, and intestinal barrier function so that linarin has the potential to be a useful dietary strategy in the therapy of inflammatory bowel disease (Table 1) [70].  Figure 3 displays the gastroprotective mechanism of linarin.

### 3.5. Metabolic Diseases and Disorders

#### 3.5.1. Diabetes

Diabetes is a chronic medical condition characterized by elevated blood glucose levels and disruptions in protein and fat metabolism. This occurs when cells are unable to effectively metabolize glucose, leading to increased blood glucose levels. This may result from insufficient insulin production by the pancreas or from cells' inability to properly utilize the insulin produced [71]. Diabetes mellitus is one of the biggest health problems the world has ever faced, and it is now becoming more and more common [72]. In 2019, 463 million people worldwide were afflicted with diabetes mellitus, often known as T2DM, one of the most prevalent chronic and avoidable ailments. The International Diabetes Federation predicts that by 2045, there will be 700 million diabetics worldwide, a 51% increase from 2019 [73]. Patients suffering from T2DM are more susceptible to many immediate and long-term outcomes cancers, microvascular disorders (nephropathy, retinopathy, and neuropathy), and macrovascular illnesses (hyperlipidemia, coronary artery disease, hypertension, strokes, peripheral vascular disease, heart attacks, and cerebral vascular disease) are among the sequelae [74].

**Figure 4:**
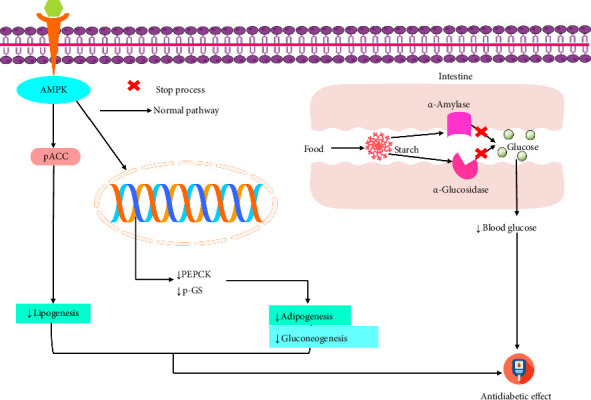
Possible antidiabetic and antiobesity mechanism of linarin [p-ACC: pH-dependent transcription factor; AMPK: AMP-activated protein kinase; PEPCK: phosphoenolpyruvate carboxykinase; p-GS: proteoglycans].

Recent in vitro and in vivo studies have shown that linarin, at concentrations of 5, 10, and 15 μmol/L in HepG2 cell lines and at doses of 25, 50, and 100 mg/kg in monosodium glutamate (MSG)–induced obese SD mice, improved metabolic parameters. Linarin treatment increased insulin tolerance, glucose tolerance, phosphorylated AMPK (p-AMPK), and phosphorylated ACC (p-ACC) levels, while reducing triglycerides, cholesterol, PEPCK, and phosphorylated GS (p-GS) [23]. An in vivo study demonstrated that linarin, at a dose of 20 mg/kg, alleviated diabetic lung injury in diabetic mice. This effect was achieved by reducing hyperglycemia, dyslipidemia, lung inflammation, and alveolar epithelial–mesenchymal transition, while enhancing the AMPK/NEU signaling pathway [34]. Furthermore, an in silico investigation demonstrated that linarin reduced T2DM by inhibiting αA and αG (Table 2) [75]. The possible antidiabatic mechanism of linarin is displayed in Figure 4.

#### 3.5.2. Hypertension

Abnormally elevated arterial blood pressure is known as hypertension or high blood pressure. According to Joint National Committee 7 (JNC7), hypertension is defined as a diastolic blood pressure of 90 mmHg or more and/or a systolic blood pressure of 140 mmHg or higher [76]. Heart attacks, strokes, renal failure, blindness, blood vessel ruptures, and cognitive decline are possible outcomes. It is estimated that 7.5 million fatalities per year, or 12.8% of all deaths worldwide, are attributed to high blood pressure [76]. According to estimates, hypertension is accountable for 57% of fatalities from stroke and 24% of deaths from coronary heart disease [77]. There will probably be 1.56 billion people on earth by 2025 suffering from hypertension [78]. One of the main cardiovascular risk factors for hypertension is cigarette smoking [79]. Overconsumption of salt in the diet (sodium chloride) is linked to a higher risk of hypertension, which is particularly significant for stroke and other cardiovascular diseases as well as renal ailments [80]. Hypertension and alcohol usage are associated with all-cause death [81].

A study by Qiaoshan et al. [29] demonstrated that linarin, administered at doses of 75 and 150 mg/kg, effectively reduced blood pressure and suppressed the renin–angiotensin system (RAS) in spontaneously hypertensive rats (SHRs) and Wistar–Kyoto (WKY) rats. Blood pressure measurements were taken using the CODA Mouse and Rat Tail-Cuff Blood Pressure System (Table 2). [29].

### 3.6. Neuroprotective Effect: Underlying Molecular Mechanisms

#### 3.6.1. Alzheimer's Disease (AD)

AD causes dementia and gradual memory loss [82]. The early stages of the condition are characterized by deficits in the capacity to encode and retain new memories. The subsequent stages are accompanied by increasing changes in thinking and acting. Neurodegeneration, synapse loss, and a decrease in synaptic strength are caused by changes in the cleavage of amyloid precursor protein (APP), the creation of the APP fragment beta-amyloid (Aβ), and hyperphosphorylated tau protein aggregation [83]. It is an incurable disease that frequently results in death [84]. AD has been related to more than 40 hereditary risk loci that have already been discovered. Heritable characteristics contribute to 60%–80% of the chance of acquiring AD. Although lifestyle factors have no direct impact on the pathophysiology of AD, they may still contribute to a better result in persons with AD [85]. Numerous possible risk factors include metal ions, oxidative stress, vascular diseases, protein dysfunctions, and changes in the populations of mitochondria influence and accelerate the long-term course of AD [86].

An in vitro study found that linarin, at concentrations of 0.1, 1.0, and 10 μM, is effective in treating AD. In PC12 cell lines exposed to Aβ_25–35_ (10–40 μM)–induced neurotoxicity, linarin improved cell viability; enhanced phosphorylation of Akt, Bcl-2, and the PI3K/Akt pathway; and reduced apoptotic cells, acetylcholinesterase (AChE) activity, and GSK-3β levels. These findings suggest that linarin inhibits neurotoxicity caused by Aβ_25–35_ and indicates its potential as a powerful anti-Alzheimer's agent through AChE inhibition and neuroprotection [22]. In a study by Feng et al. [87], in vitro experiments showed that linarin significantly inhibited AChE activity, with an IC50 value of 3.801 ± 1.149 μM, measured using Ellman's colorimetric method on mouse brain tissue. Additionally, an ex vivo investigation revealed that mice administered linarin intraperitoneally at doses of 35, 70, and 140 mg/kg had significantly reduced AChE activity in the cortex and hippocampus. These findings suggest that linarin has strong AChE inhibitory effects, indicating its potential as a therapeutic option for AD [87]. Another in vitro study by Oinonen et al. [88] demonstrated that linarin effectively reduced AChE activity (Table 3).

#### 3.6.2. Ischemia–Reperfusion

Ischemia–reperfusion injury is a complicated pathological process that starts with tissue anoxia and is followed by the generation of inflammatory reactions brought on by free oxygen radicals [91]. Because of anaerobic metabolism and lactate buildup after chronic ischemia, intracellular pH and ATP levels drop. This leads to the malfunctioning of ATPase-dependent ion transport pathways, which in turn causes a rise in intracellular and mitochondrial calcium levels (calcium overload), cell rupture and swelling, and necrotic, apoptotic, necroptotic, and autophagic processes that cause cell death. Reperfusion restores oxygen levels, but not before proinflammatory neutrophils enter ischemic tissues and produce a spike in ROS, worsening ischemic damage [92]. An extensive spectrum of ailments, including as myocardial infarction, ischemic stroke, acute renal damage, trauma, circulatory arrest, sickle cell disease, and sleep apnea, are associated with increased morbidity and mortality due to ischemia and reperfusion-induced tissue damage [93].

Linarin, at concentrations of 3.3, 10, and 30 μM, demonstrated a significant cardioprotective effect in H9C2 cells (in vitro). The protective mechanisms included reducing apoptosis rate, hypoxia–reoxygenation injury, and cytochrome C levels, while increasing Nrf-2, PI3K/Akt signaling, and NF-kB activity. This study suggests that linarin may activate Nrf-2 to protect cardiac tissue from ischemia–reperfusion injury [24]. Another in vivo inquiry by Xie et al. [19] stated that linarin (4, 20, and 40 mg/kg) reduced MCAO-induced ischemic stroke of C57BL6/j male mice by downregulating infarct volume, neural cell apoptosis, and inflammatory response and by upregulating neurological function, CSPG4, extracellular matrix, KDELR, phosphorylation of mitogen-activated protein kinases, and cerebral blood flow (Table 3) [19].

### 3.7. Sedative Effect and Anticonvulsant

A prevalent medical disorder called insomnia can be demonstrated by difficulty falling asleep or staying asleep, along with symptoms like restlessness or discomfort when awake [94]. Twenty percent of adults have occasional symptoms of insomnia. Insomnia is more common in women, older persons, and those from low-income backgrounds [95]. There is a strong correlation between insomnia and a higher chance of depression, an increased risk for dementia, and cardiovascular disease-related death [96–98]. For critically ill patients, sedation is mostly used to relieve pain and distress [99]. Adult patients in the medical critical care unit who have respiratory failure and need mechanical ventilation must be sedated [100]. Dexmedetomidine is a highly selective α2 adrenergic receptor agonist of the current generation that offers several benefits, such as sedative and analgesic effects [101]. Young children and elderly individuals are disproportionately affected by convulsive status epilepticus (SE), a frequent neurological emergency. In children, SE is linked to a mortality rate of about 0%–3%. In elderly persons and survivors, 20%–30% frequently have neurological and cognitive deficits [102].

A recent study by Kim et al. [89] reported that linarin (5 and 10 mg/kg) improved sleep quality in pentobarbital-induced sleep in mice by enhancing Cl− influx via GABA_A_ receptors, thereby increasing sleep duration and reducing sleep onset time. Following linarin administration, levels of GAD65/67 and GABA_A_ receptor subunits α1 and β2 in the hippocampus, frontal cortex, and hypothalamus were analyzed. The activation of Cl− channels is suggested to be responsible for the observed effects on sleep enhancement [89]. Another investigation by Nugroho et al. [31] stated that linarin exerted both anticonvulsant and sedative. Linarin at doses of 10 and 20 mg/kg diminished the PTZ-induced convulsion assay in mice and also 10 and 20 mg/kg significantly diminished pentobarbital-induced sleep in mice by increasing sedative effect, anticonvulsant effect, and sleep duration and by decreasing CNS excitation and sleep onset (Table 3) [31].

### 3.8. Depression

One of the most incapacitating mental disorders in the world is depressive disorder [103]. As of right now, we understand that depression is associated with a low-grade, persistent inflammation response, immunity mediated by cells, and an activated compensatory anti-inflammatory reflex system [104]. Comorbid anxiety and depression, sometimes known as anxious depression, is a rather frequent syndrome, despite the fact that anxiety and depression have historically been treated as separate conditions under the diagnostic criteria [105]. All things considered, the evidence is compelling enough to propose that depression in later life should be seen as a precursor to dementia and that depression in childhood may work as a risk factor for dementia in later life [106]. One in four patients with T2DM has clinically severe depression. In turn, depression improves the possibility of acquiring T2DM, which in turn elevates the risk of insulin resistance, hyperglycemia, and micro- and macrovascular issues, and vice versa [107].

**Figure 5:**
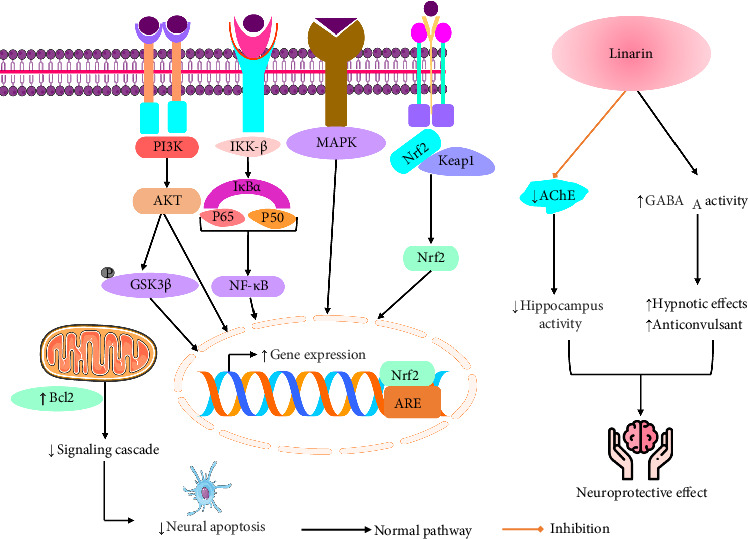
Possible neuroprotective mechanism of linarin [PI3K: phosphatidylinositol 3-kinases; IKKβ: IκB kinase β; AKT: protein kinase B; P65: transcription factor P65; P50: transcription factor P50; NF-κB: nuclear factor kappa-light-chain-enhancer of activated B cells; IκBα: nuclear factor of kappa light polypeptide gene enhancer in B-cell inhibitor, alpha; Bcl2: B-cell leukemia/lymphoma 2 protein; GSK3β: glycogen synthase kinase 3β; Nrf2: nuclear factor erythroid 2–related factor 2; Keap1: Kelch-like ECH-associated protein 1; AChE: acetylcholinesterase; GABA_A_: γ-aminobutyric acid type A].

An investigation by Guzmán-Gutiérrez et al. [90] stated that linarin (30 and 60 mg/kg) produced an antidepressant effect on mice. Test run by the procedure of FST in mice (forced swim test) (Table 3) [90]. The possible neuroprotective mechanism of linarin is illustrated in Figure 5.

### 3.9. Anticancer

#### 3.9.1. Induction of Oxidative Stress

Oxidative stress and cell damage are caused by free radical oxidants, which include ROS, reactive nitrogen species, and reactive sulfur species [108]. Hydrogen peroxide (H_2_O_2_), superoxide (O_2_), and hydroxyl radicals (HO) are examples of ROS. High level of ROS induces the DNA damage and trigger programmed cell death [109].

A recent study demonstrated that the combination therapy with TRAIL and linarin also significantly increased the generation of ROS, up to 39.86 ± 2.32%, and concurrently elevated JNK phosphorylation. By inducing apoptosis through ROS formation, linarin with TRAIL may offer a beneficial approach against TRAIL-resistant glioma cells (Table 4) [110].

#### 3.9.2. Cytotoxicity

Cytotoxic substances cause permanent changes in cells and tissues, including cell death, when they come into contact with living things. They also deteriorate cell functioning [111]. Several kinds of malignancies are treated with DNA-damaging medications as first-line therapy, including alkylating compounds, cytotoxic antibiotics, and DNA topoisomerase poisons [112].

A study by Xu et al. [110] found that a noncytotoxic dose of linarin (5 μM) significantly enhanced TRAIL-induced cytotoxicity (80 ng/mL), leading to increased cell death (Table 4) [110].

#### 3.9.3. Apoptotic Effect

Apoptosis refers to the mechanism by which cells undergo intentional self-destruction. Apoptosis is involved in the prevention of cancer [113]. When cells undergo DNA breakage, P53 and P21 are upregulated, resulting in an increased BAX protein level [114, 115]. The BAX protein triggers cytochrome C release by decreasing mitochondrial membrane potential. However, the cytochrome C release depends on BCL2. Downregulation of BCL2 increases cytochrome C release [116]. After that, cytochrome C activates the caspase protein, and cells undergo an apoptosis process [117]. In addition, DNA damage increases the PARP protein level, which directly activates the caspase protein, resulting in cancer cell apoptosis [118]. On the other hand, FADD is a protein adapter that joins the death-inducing signaling complex (DISC) in response to death receptors being activated. FADD serves as a shared pathway in both CD95-induced and TNF-R-induced apoptosis [119].

**Figure 6:**
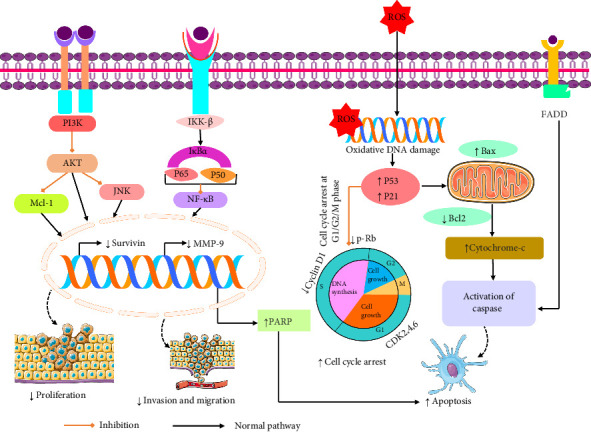
Possible anticancer mechanism of linarin [PI3K: phosphatidylinositol 3-kinases; Mcl-1: myeloid cell leukemia sequence 1; AKT: protein kinase B; IKKβ: IκB kinase β; IκBα: nuclear factor of kappa light polypeptide gene enhancer in B-cell inhibitor, alpha; P65: transcription factor P65; P50: transcription factor P50; NF-κB: nuclear factor kappa-light-chain-enhancer of activated B cells; JNK: Jun N-terminal kinase; MMP-9: matrix metalloproteinase; ROS: reactive oxygen species; p-RB: retinoblastoma protein; FADD: Fas-associated protein with death domain; Bax: Bcl-2-associated X protein; Bcl2: B-cell leukemia/lymphoma 2 protein; CDK: cyclin-dependent kinase; P21: cyclin-dependent kinase inhibitor; P53: tumor protein; PARP: poly(ADP-ribose) polymerase].

Numerous investigations have shown that linarin has apoptosis properties. A study by Zhen et al. [120] showed that linarin (10–100 μM) induces apoptosis through the upregulation of p53, p21, PARP, Bax, and caspase-3 protein expression in brain cancer cells [120]. In a prior study in 2005, Singh and his team revealed that linarin (25–100 mM) blocks prostate cancer cells development by upregulating PARP, and cells go through the apoptosis process [121]. Another study exhibited that linarin (2.5–10 mM) accelerated the apoptosis process via the elevation of caspase-8/caspase-9/caspase-3, PARP cleavage, and Cyto-c levels, as well as the alleviation of Bcl-2 protein levels. Additionally, linarin activated the apoptosis process through the activation of death receptors (DR-4 and DR-5) and the initiation of adaptor proteins (Table 4) [110] (Figure 6). Therefore, linarin has apoptosis activity through the activation of death receptors and caspase proteins.

#### 3.9.4. Antiproliferative Effect

The growth and spread of cancer depend on proliferation. The noted alterations in the expression and/or functioning of proteins associated with the cell cycle provide evidence for this phenomenon [122]. In many biological activities, such as cell development, proliferation, and survival, the consequence of various extracellular signals is facilitated by the phosphatidyl inositol 3-kinase (PI3K)/Akt pathway. The modification of this pathway's integrants by mutation of its coding genes raises the signaling and can therefore result in cellular alteration [123]. Human health depends on NF-κB, and abnormal activation of NF-κB promotes the growth of a number of autoimmune, inflammatory, and malignant tumors [124]. A nuclear transcription factor that promotes apoptosis is called p53. The p53 gene has been regarded as one of the classical type tumor suppressors as mutations causing loss of function have been found in over 50% of human malignancies [125]. Control of eukaryotic cell proliferation depends on the proper cell cycle transition from G1 phase to S phase. During the G1 phase, growth-dependent cyclin-dependent kinase (CDK) activity promotes DNA replication and initiates the G1-to-S phase transition [126].

A study by Seo et al. [127] stated that linarin (0.1–10 mg/mL) inhibited cell proliferation by inhibiting PI3K/Akt pathway, S phase, and G1 phase and by inducing CDK inhibitor p27 (Kip1) and prevented lung cancer [127]. A different study revealed that linarin (10, 20, 40, 80, and 100 μM) inhibited the growth of cells via upregulating p53 and downregulating NF-κB/p65 (Table 4) [120].

#### 3.9.5. Inhibition of Cell Migration and Invasion

Individual and collective cell migration are the two main forms of cancer cell invasion. These patterns enable tumor cells to multiply into surrounding tissues and break through extracellular matrix barriers [128]. There are also more processes via which cancer cells might spread and move, such as mesenchymal cell migration, and amoeboid cell migration [129]. It is well established that the synthesis of MMP-9 promotes tumor cell invasion and metastasis [110]. The cytoplasmic presence of NF-κB is maintained by NFKB inhibitor α (IκB-α), which functions as a negative regulator of the conventional NF-κB pathway [130].

A study by Jung et al. [27] established that linarin at concentration of 5, 10, 50, 100, 200, and 500 μM significantly decreased the IR-induced cell migration and invasion via downregulating MMP-9 pathway and suppressing NF-κB and IκB-α phosphorylation in order to reduce NF-κB activity (Table 4) [27].

## 4. Clinical Evidence

Clinical studies in humans have been improved to explain the safety and effectiveness of NPs [131]. Clinical studies using NPs (such as flavonoids and saponins) have been found in recent years [132]. According to some researchers, the benefits of flavonoids are still supportive for usage, but clinical guidelines and reviews have been less enthusiastic about them [133, 134].

In 2018, a clinical study of flavonoids for the treatment of hemorrhagic disease in male and female patients was overseen [135]. In some literature, it has been shown to reduce relapse and attenuate bleeding [136, 137]. In the study by Corsale et al. [135], patients with Golligher's grade and piles were evaluated, although the specific flavonoid administered was not disclosed. The control group received a mixture of diosmin combined with other flavonoids, including linarin. Results showed that bleeding improved in both the study and control groups after 1 and 6 months, with no acute complications reported, although gastrointestinal side effects were noted. The most favorable outcomes were observed at the 6-month mark for both groups [136].

## 5. Toxicological Profile

Toxicity testing in drug discovery involves assessing the potential adverse effects of newly developed compounds on living organisms [138]. These tests are crucial in identifying any harmful effects that could arise from drug candidates before they advance to clinical trials [139]. By evaluating toxicity early in the drug discovery process, researchers can prioritize safer compounds and optimize their therapeutic potential while minimizing risks to patients [140]. Various approaches both in vivo and in vitro are utilized to assess toxicity, including cell culture assays, animal models, and computational modeling techniques. The toxicological effects of flavone glycosides, like any other compound, depend on various factors, including dosage, duration of exposure, individual sensitivity, route of exposure, metabolism, interaction, and target organism [141, 142].

Research exhibited that linarin induces cytotoxicity in cell lines (A-172, U343, U87MG, T98G) at doses ranging from 2.5 to 10 μM in vitro study [110]. On the other hand, an acute toxicity test of Chrysanthemum indicum plant showed no mortality and behavioral transformation of animals at dose ranging from 0.5 to 4 g/kg where the GC–MS analysis found the linarin compound. Additionally, the HPLC test demonstrated the highest amounts of linarin compound, which is 48.3 mg/g. This study exhibited that the maximal tolerance dose (MTD) of the test sample was asserted larger than 4 g/kg in mice [50].

## 6. Conclusion

NPs serves as an unending resource for drug development, new chemotypes and pharmacophores, framework for drug amplification into potent medication for a wide variety of therapeutic purposes, and other useful chemical compounds. The goal of the current research was to assess linarin's potential for therapeutic use by evaluating the existing evidence from different preclinical trials and the fundamental mechanisms that give rise to these consequences. The results showed that linarin has a large number of pharmacological activities, including anti-inflammatory, osteoprotective, hepatoprotective, antidiabetic, gastroprotective, lung protective, cardioprotective, and also neuroprotective effects. Our findings also demonstrate that linarin exhibits remarkable anticancer potentials via a range of molecular mechanisms, like apoptotic cell death, oxidative stress induction, genotoxic effect, cytotoxic effect, antiproliferative effect, and reduction of cancer cell invasion and migration against a variety of malignancies, including lung, brain, prostate, and glioma cell cancers. PK investigations indicated that linarin is efficiently absorbed and dispersed throughout the body's organs; however, there was a report of low oral bioavailability of the compound. Therefore, the finding of an alternate path is required to enhance the bioavailability and effectiveness of linarin. Relying on the overall results, we propose that more comprehensive investigations and trials are essential to establish the compound as an effective medicinal agent in the treatment of different disorders.

## Figures and Tables

**Table 1:** Linarin's biological activity based on database reports from preclinical studies.

Related disease/effect	Test medium/cell line/test system	Dose/concentration/IC50 (R/A)/course interval	Possible mechanism	Reference
Anti-inflammatory effect	RAW264.7, in vitro	5, 10, 20, and 30 μM	↓NO, ↓ IL-1β, ↓ IL-6, ↓Phagocytic ability of macrophages, ↓ MHC II, ↓CD80	[26]
	LPS-induced (HUVECs) human umbilical vein endothelial cells, in vitro	10 and100 μmol/L	↓NO, ↓IL-1β, ↓TNF-α	[45]
	RAW 264.7, in vitro	2.5, 5, 10, and 20 μg/mL	↑Cytokines, ↑TNF, ↑IL-1, ↓NO	[46]
	RAW264.7, in vitro	40, 80, and 160 μM	↓NO, ↓TNF-α, ↓IL-1β, ↓IL-6, ↓mRNA levels, ↓iNOS, ↓NF-κB p65, ↓IκBα, ↓ERK, ↓JNK, ↓p38, ↓Akt, ↓MAPKs	[47]
	RAW264.7, in vitro	20, 40, and 80 μM	↓NO, ↓TNF-α, ↓IL-6, ↓PGE2	[48]
	LPS-induced NO release in macrophages, in vitro	8–32 µM	↓NO release	[49]
	Xylene-induced mouse ear edema Acetic acid–induced mouse vascular permeability, carrageenan-induced mouse hind paw edema, and cotton pellet–induced rat granuloma formation, in vivo	40, 80, and 120 mg/kg for mouse; po 20, 40, and 60 mg/kg for rats; po	↓ROS, ↓NF-*κ*B signaling, ↓iNOS, ↓COX-2, ↓TNF-*α*, ↓IL-1*β*, ↓IL-6, ↓PGE2, ↓MDA level, ↑SOD, ↑GPx, and ↑GRd	[50]

Osteoporosis	Cadmium-induced osteoporosis in mice, in vivo	20 and 40 mg/kg (po)	↑SOD, ↑CAT, and ↑GPx levels, ↓MDA, ↓Lipid peroxidation, ↓RANKL mRNA levels, ↓NF-κB p65, IKKβ, ↓IL-6, ↓TNF-α, ↓RANK/RANKL/OPG pathway, ↓Oxidative stress	[25]
	RAW 264.7, in vitro	1–20 and 1–20 μM	↓RGD peptide, ↓Paxillin, ↓Gelsolin, ↓Cdc42, ↓CD44, ↓Osteoclast function	[56]
	RANKL-induced osteoclastogenesis in C57BL/6 mice, in vivo	0.1, 1, and 10 mg/mL	↓NFATc1, ↓TRAP, ↓OSCAR, ↓c-Fos, ↓NF-kB p65, ↓NF-kB signaling pathway	[18]
	MC3T3-E1, in vivo	0.2–5 mg/mL	↑cell survival, ↑ALP activity, ↑Collagen, ↑Calcium deposition, ↑Osteocalcin secretion, ↓RANKL, ↓PCO, ↓MDA, ↓Oxidative damage	[28]

Osteoarthritis	LPS-induced human OA chondrocytes, in vitro	3.75, 7.5, 15, and 30 μM	↓NO, ↓PGE2, ↓IL-6, ↓TNF-α, ↓COX-2, ↓iNOS, ↓TLR4, ↓MD-2, ↓NF-κB pathway	[33]
	DMM mice, *n* = 15, in vivo	30 mg/kg (po)		
	SW1353, in vitro	0.5, 1, 5, 10, 50, and 100 μg/mL	↓SOX9, ↓ADAMTS-4, ↓ADAMTS-5	[59]

Postmenopausal osteoporosis	MC3T3-E1, in vitro	0.1, 1, and 10 µM	↑Osteoblast proliferation, ↑Differentiation in MC3T3-E1, ↑ALP activity, ↑Mineralization of extracellular matrix, ↑OCN, ↑BSP, ↑COL-I, ↑Phosphorylation of SMAD1/5, ↑PKA, ↑BMP-2/RUNX2 pathway, ↓Serum levels of ALP and OCN	[32]
	OVX-induced osteoporosis in mice, in vivo, *n* = 10	50 and 150 mg/kg		

Fulminant hepatic failure	GalN/LPS-induced hepatic failure in male ICR mice, in vivo	12.5, 25, and 50 mg/kg (po)	↓Fas-associated death domain, ↓Caspase-8, ↓Cytochrome c, ↓Caspase-3, ↓Pro-apoptotic Bim phosphorylation, ↓TNF-α-mediated apoptotic pathway, ↑Bcl-xL, ↑ phosphorylation of STAT3	[35]

Liver injury	(NASH) induced HFHC in male Sprague–Dawley rats. *N* = 10, in vivo	15, 30, and 60 mg/kg (po)	↑SCD1, ↓ALT, ↓AST, ↓c-JNK, ↓MCP-1, ↓TNF-*α, ↓*CXCL1, ↓Lobular inflammation, ↓Liver cholesterol.	[66]
	HepG2, in vitro	12.5, 25, and 50 μg/mL	↓ROS, ↓MAPKs phosphorylation, ↑Keap1, ↑ALT, ↑AST, ↑Free Nrf2, ↑Phase II detoxification enzymes	[68]

Diabetic liver injury	High-glucose and high-palmitic acid–induced hepatocyte injury in mice, in vivo	15, 30, and 60 mg/kg/day	↓Hepatic lipid accumulation, ↓Oxidative stress, ↓Inflammation, ↓AKR1B1 ↓Apoptosis	[36]
	Rat liver tissues in mice, in vitro	20 μM		

Acute liver injury	Carbon tetrachloride (CCl4)–induced male BALB/c mice model, in vivo	12.5, 25, and 50 mg/kg once daily for 7 days	↓ROS-induced oxidative stress, ↓TLR4/MyD88 and JNK/p38/ERK-mediated inflammatory responses, and ↑Beclin 1/LC3II-mediated autophagic flux	[67]

Inflammatory bowel disease	Dextran sulfate sodium (DSS)–induced colitis in C57BL/6J mice, in vivo	25 and 50 mg/kg (po)	↑Histopathological damage, ↑Mucosal layer, and intestinal barrier function, ↓IL-6, ↓TNF-α, ↓IFN-γ, ↓IL-1β, ↓Myeloperoxidase	[70]

*Note:* ↑: increase/stimulation/upregulation; ↓: decrease/inhibition/downregulation; MCP-1: monocyte chemotactic protein-1; IL-6: interleukin-6; IFN-γ: interferon-gamma; IL-1β: interleukin-1beta; MHC II: major histocompatibility complex class two; CD80: cluster of differentiation 80; IL-1: interleukin-1; NF-κB p65: nuclear factor kappa-light-chain-enhancer of activated B cells (p65); AKR1B1: Aldo-keto reductase family 1 member B; IκBα: nuclear factor of kappa light polypeptide gene enhancer in B-cell inhibitor, alpha; JNK: Jun N-terminal kinase; ERK: extracellular signal–regulated kinase; Akt: protein kinase B; MAPKs: mitogen-activated protein kinase; PGE2: prostaglandin E2; TNF-α: tumor necrosis factor-alpha; COX-2: cyclooxygenase 2; MDA level: malondialdehyde; (SOD, GPx, GRd): antioxidants; CAT: chloramphenicol acetyltransferase; IKKβ: IκB kinase β; RANK: receptor activator of nuclear factor κB; OPG: osteoprotegerin; RGD: arginyl-glycyl-aspartic acid; cdc42: cell division control protein 42; CD44: cell surface adhesion receptor; NFATc1: nuclear factor of activated T cells 1; Bcl-xL: cell lymphoma-extra-large; OSCAR: osteoclast-associated receptor; ALP: alkaline phosphatase; PCO: protein carbonyl; TLR4: Toll-like receptor 4; RANKL: receptor activator of nuclear factor-kB ligand; MD-2: myeloid differentiation protein-2; SOX9: transcription factor; ADAMTS-4,5: a disintegrin and metalloproteinase with thrombospondin motifs; OCN: osteocalcin; BSP: bone sialoprotein; COL-I: Type I collagen; PKA: protein kinase A; BMP-2: bone morphogenetic protein-2; RUNX2: Runt-related transcription factor 2; STAT3: signal transducer and activator of transcription 3; SCD1: stearoyl-CoA desaturase; ALT: alanine aminotransferase; AST: aspartate aminotransferase; c-JNK: c-Jun N-terminal kinase; CXCL1: chemokine (C-X-C motif) ligand 1; Keap1: Kelth-like ECH-associated protein; Free Nrf2: nuclear factor (erythroid-derived 2)–like 2.

Abbreviations: iNOS, inducible nitric oxide synthase; NO, nitric oxide; ROS, reactive oxygen species; TRAP, tartrate-resistant acid phosphatase.

**Table 2:** Lidarin's efficacy against metabolic disorders in a number of preclinical and nonclinical testing methods.

Related disease/effect	Test medium/cell line/test system	Dose/concentration/IC50 (R/A)/course interval	Possible mechanism	Reference
Diabetes	HepG2, in vitro	5, 10, and 15 lmol/L	↑Insulin tolerance, ↑Glucose tolerance, ↑ p-AMPK, ↑p-ACC, ↓Triglyceride, ↓Cholesterol, ↓PEPCK, and ↓p-GS	[23]
	Obesity induced in SD mice via monosodium glutamate (MSG) (sc), *n* = 10, in vivo	25, 50, and 100 mg/kg (po)		

Diabetic lung injury	In vivo, diabetic mice	20 mg/kg	↓Hyperglycemia, ↓Dyslipidemia, ↓Lung inflammation in diabetic mice, ↓Alveolar epithelial–mesenchymal transition, ↑AMPK/NEU-mediated signaling pathway	[36]

Type 2 diabetes mellitus	*In silico*		↓αA and ↓αG	[75]

Hypertension	SHR WKY rat, CODA mouse, and rat tail-cuff blood pressure system.	75 and 150 mg/kg	↓Blood pressure, ↓RAS	[29]

*Note:* ↑: increase/stimulation/upregulation; ↓: decrease/inhibition/downregulation; p-AMPK: AMP-activated protein kinase; p-ACC: pH-dependent transcription factor; PEPCK: phosphoenolpyruvate carboxykinase; p-GS: proteoglycans; αA: α-amylase; αG: α-glucosidase.

Abbreviation: RAS, renin–angiotensin system.

**Table 3:** Neuroprotective effects of linarin and their mechanisms.

Related disease/effect	Test medium/cell line/test system	Dose/concentration/IC50 (R/A)/course interval	Possible mechanism	Reference
Alzheimer's disease	Aβ_25-35_-induced neurotoxicity in cultured rats (PC12 cells)	0.1, 1.0, and 10 μM	↑Cell viability, ↓Apoptotic cells, ↓Acetylcholinesterase activity, ↑Phosphorylation of Akt, ↑Bcl2, ↑ PI3K/Akt. ↓GSK-3β	[22]
	Mice's brain, in vitro.	IC_50_ of 3.801 ± 1.149 μM	↓AChE, ↓Hippocampus activity	[87]
	Huperzine-induced (AD) male Kunming (KM) mice (i.p.), *n* = 6, in vivo	35, 70, and 140 mg/kg (i.p.).		
	AChE (EC 3.1.1.7, Type VI-S from electric eel)		↓AChE	[88]

Ischemia–reperfusion	H9C2, in vitro	3.3, 10, and 30 μM	↑Nrf-2, ↑PI3K/Akt signaling pathway, ↑ NF-kB, ↓Cytochrome C, ↓Apoptosis rate, ↓Hypoxia–reoxygenation	[24]

Ischemic stroke	MCAO-induced ischemic stroke of C57BL6/j male mice, in vivo *n* = 10	4, 20, and 40 mg/kg (i.p.)	↑ Neurological function, ↑CSPG4, ↑Extracellular matrix. ↑KDELR, ↑Phosphorylation of mitogen-activated protein kinases, ↑Cerebral blood flow. ↓Infarct volume, ↓Neural cell apoptosis, ↓Inflammatory response.	[19]

Sedative	Male ICR mice, pentobarbital-induced sleep in male ICR mice, in vivo, *n* = 10	5 and 10 mg/kg (i.p.)	↑Cl^−^ influx via GABA_A_, ↑Hypnotic effects, ↓Sleep onset, ↑Sleep duration	[89]

Sedative and anticonvulsant	PTZ-induced convulsion in mice, in vivo	10 and 20 mg/kg	↑Sedative, ↑Anticonvulsant, ↑Sleep duration, ↓ sleep onset, ↓CNS excitation	[31]
	Pentobarbital-induced sleep, in vivo (i.p.), *n* = 6	10 and 20 mg/kg		

Depression	FST in mice (forced swim test)	30 and 60 mg/kg	↓Depression	[90]

*Note:* ↑: increase/stimulation/upregulation; ↓: decrease/inhibition/downregulation; Bcl2: B-cell lymphoma 2; GSK-3β: glycogen synthase kinase-3 beta; KDELR: KDEL receptor; GABA_A_: γ-aminobutyric acid type A; PI3K/Akt: phosphatidylinositol 3-kinase/protein kinase B.

**Table 4:** Anticancer effects of linarin with underlying mechanisms.

Related disease/effect	Test medium/cell line/test system	Dose/concentration/IC50 (R/A)/course interval	Possible mechanism	Reference
Glioma (malignant brain cancer)	U87-MG, U251, HEB, HL-7702, H4, A172, BV2, in vitro	10, 20, 40, 80, and 100 μM	↑Apoptosis, ↑p21, ↑Bax, ↑Caspase-3, ↑p53, ↑PARP, ↓Proliferation of glioma, ↓Kappa-B (NF-κB)/p65, ↓Survivin, ↓p-Rb, ↓CyclinD1, ↓ Glioma progression	[120]
	Male BALB/c nude mice, xenograft tumor, in vivo	12.5, 25, and 50 mg/kg		

Prostate cancer	LNCaP, DU145, in vitro	25–100 μM	↑G1 arrest, ↑Cip1/p21, ↑Poly-(ADP-ribose), ↑Apoptosis, ↓CDK2, ↓CDK4, ↓CDK6	[121]

Lung cancer	A549, in vitro IR-induced lung cancer	5, 10, 50, 100, 200, and 500 μM, IC50 = 282 μM	↓Cancer cell migration and invasion, ↓MMP-9 pathway, ↓NF-κB, ↓IκB-α phosphorylation	[27]

Glioma cell cancer	A-172, U343, U87 MG, T98G, in vitro	2.5, 5, and 10 μM	↑Cytotoxicity, ↑Apoptosis, ↑Caspase-8/-9/-3, ↑PARP cleavage, ↑Cyto-c, ↑FADD, ↑DISC, ↑DR-4 and DR-5, ↑ROS, ↓JNK phosphorylation, ↓Tumor growth, ↓Bcl-2, ↓Mcl-1, ↓Survivin. ↓Anti-apoptotic protein	[110]
	Male C57BL/6J mice, TRAIL-induced tumor growth in xenograft, in vivo	25 mg/kg		

Lung cancer	A549, CCL-185, in vitro	0.1–10 mg/mL	↑p27^Kip1^, ↓Cell proliferation, ↓Phosphatidylinositol 3-kinase/Akt pathway, ↓Akt, ↓S phase, ↓G1 phase	[127]

*Note:* ↑: increase/stimulation/upregulation; ↓: decrease/inhibition/downregulation; Bax: Bcl-2-associated X protein; PARP: poly(ADP-ribose) polymerase; FADD: Fas-associated protein with death domain; Bcl-2: B-cell leukemia; Mcl-1: myeloid cell leukemia sequence 1.

Abbreviations: CDK, cyclin-dependent kinase; DISC, death-inducing signaling complex; DR, death receptor; MMP, matrix metalloproteinase.

## Data Availability

Data will be made available upon request.
